# Cytokine patterns in nasal secretion of non-atopic patients distinguish between chronic rhinosinusitis with or without nasal polys

**DOI:** 10.1186/s13223-016-0123-3

**Published:** 2016-04-27

**Authors:** Katrin König, Christine Klemens, Mareike Haack, Marion San Nicoló, Sven Becker, Matthias F. Kramer, Moritz Gröger

**Affiliations:** Department of Otorhinolaryngology, Head and Neck Surgery, University Medical Center Großhadern of the Ludwig-Maximilians-University Munich, Marchioninistr. 15, 81377 Munich, Germany; Department of Otorhinolaryngology, Head and Neck Surgery, University Medical Center of the Johannes Gutenberg University Mainz, Langenbeckstr. 1, 55101 Mainz, Germany

**Keywords:** Chronic rhinosinusitis, Nasal polyps, Nasal discharge, Mediators, Cytokines, Chemokines

## Abstract

**Background:**

Being one of the most common nasal diseases, chronic rhinosinusitis (CRS) is subdivided into CRS with nasal polyps (NP) and CRS without nasal polyps (CRSsNP). CRSsNP presents itself with a T_H_1 milieu and neutrophil infiltration, while NP is characterised by a mixed T_H_1/T_H_2 profile and an influx of predominantly eosinophils, plasma cells and mast cells. For the purpose of discovering disease-specific cytokine profiles, the present study compares levels of mediators and cytokines in nasal secretions between CRSsNP, NP, and healthy controls.

**Methods:**

The study included 45 participants suffering from NP, 48 suffering from CRSsNP and 48 healthy controls. Allergic rhinitis constituted an exclusion criterion. Nasal secretions, sampled using the cotton wool method, were analysed for IL-4, IL-5, IL-10, IL-12, IL-13, IL-17, IL-8, GM-CSF, G-CSF, IFN-γ, MCP-1, MIP-1α, MIP-1β, eotaxin, and RANTES, and for ECP and tryptase, using Bio-Plex Cytokine assay or ELISA, respectively.

**Results:**

Elevated levels of IL-5, IL-17, G-CSF, MCP-1, MIP-1α, MIP-1β, ECP, and tryptase, as well as decreased levels of IL-10, IL-12, IL-13, and IFN-γ were detected in NP. CRSsNP presented increased levels of RANTES and MIP-1β while IL-13 was decreased. No differences between the three groups were found for IL-4, IL-8, GM-CSF, and eotaxin.

**Conclusions:**

The present work suggests a disequilibrium of T_H_1 and T_H_2, together with a down-regulation of regulatory T lymphocytes and up-regulated T_H_17 in NP. Moreover, elevated levels of diverse mediators represent the activation of various inflammatory cells in this disease entity. The inflammation in CRSsNP, however, is only weakly depicted in nasal secretions. Therefore, cytokines in nasal secretions may provide helpful information for differential diagnosis.

## Background

Chronic rhinosinusitis (CRS) is one of the most common nasal diseases, affecting 5 % of the Canadian population and 11 % of Europeans [1, 2]. Deteriorating both physical and mental health, CRS significantly impairs patients’ quality of life and imposes immense costs on the public health system [3, 4]. CRS is characterised by an inflammation of the nose and paranasal sinuses for more than 12 weeks, causing nasal obstruction and discharge, facial pain, and reduction of smell [5]. By nasal endoscopy, this disease is subdivided into CRS with nasal polyps (NP) and CRS without nasal polyps (CRSsNP) [5].

Nasal polyps manifest themselves macroscopically as grey masses, prolapsing into the nasal cavity. In histological sections, oedema, pseudocysts, and a colourful infiltrate of inflammatory cells are seen. In contrast, CRSsNP is characterised by fibrosis and basement membrane thickening [6].

The pathophysiology of CRS is not yet well understood. Although CRS may be associated to genetic or systemic diseases such as cystic fibrosis or sarcoidosis, the majority of the CRS patients seems to suffer from idiopathic disease [7]. Concerning the aetiopathology, local and systemic host factors as well as environmental factors have been discussed [8]. However, hypotheses about impaired innate immunity, fungi, or superantigens remain to be verified. Atopic diseases are more frequent in CRS patients than in the general population, and allergy as an associated or deteriorating factor has also been discussed [9]. Yet, a definitive answer is owing.

According to reported cell and cytokine patterns, CRSsNP and NP seem to be different disease entities. CRSsNP is characterised by a T_H_1 milieu and neutrophils. NP, on the other hand, shows a mixed T_H_1/T_H_2 profile with increased numbers of eosinophils, plasma cells and mast cells [6, 10]. However, this only applies to the majority of the Caucasian NP patients; Asian NP patients have been reported to show a T_H_1/T_H_17 polarisation, while the T cell patterns of CRSsNP were similar in both races [6, 11, 12].

The present work compares cytokines in nasal secretions of NP and CRSsNP patients to those of healthy subjects. In the present study, we wanted to study CRS in pure form. As interference between the pathophysiological processes of allergic rhinitis and CRS is conceivable, allergy testing was performed to exclude allergic patients from the study. Levels of cytokines were investigated in order to determine whether the pathophysiology of CRS is depicted in nasal secretions. Our study focusses on two major aspects: the regulation of the T cell subsets T_H_1, T_H_2, T_H_17, and regulatory T cells (T_reg_), represented by levels of interleukin (IL)-4, IL-5, IL-10, IL-12, IL-13, IL-17, and Interferon (IFN)–γ, and the regulation and activation of inflammatory cells such as granulocytes and mast cells by levels of IL-5, IL-8, granulocyte–macrophage colony-stimulating factor (GM-CSF), granulocyte colony-stimulating factor (G-CSF), eotaxin, “regulated on activation, normal T cell expressed and secreted” (RANTES) protein, macrophage inflammatory protein (MIP)-1α, MIP-1β, monocyte chemotactic protein-1 (MCP-1), eosinophil cationic protein (ECP), and tryptase.

## Methods

### Study population

141 volunteers (64 males, 77 females, mean age 41 ± 15 years) participated in the present study. Clinical history was taken by one of the investigators. All subjects were tested for allergy to aeroallergens with the in vitro allergy screening test Sx1 (Phadia, Freiburg, Germany). Based on a fluorescence-enzyme-immunoassay (FEIA) this method tests for IgE to inhalant allergens in participants’ sera. Volunteers presenting a history of allergy or a positive Sx1 were excluded from the study.

Any medication concerning the nasal disease during 6 weeks prior to the examination constituted an exclusion criterion, particularly anti-inflammatory medication such as topical nasal steroids. To detect nasal polyps and exclude patients with signs of purulent rhinitis, nasal endoscopy was performed in all volunteers. For ethical reasons, X-ray computed tomography (CT) scanning was only performed if indicated for medical care, but not for the purpose of this study.

NP (n = 45) was determined by the patient’s history and the presence of endoscopically visible polyps in the nasal cavity, the paranasal sinuses, or both.

CRSsNP (n = 48) was determined clinically by typical complaints in the patient’s history such as midfacial pain or pressure, postnasal drip, nasal obstruction, or reduction of smell. Inspection of the nose and nasal endoscopy revealed the picture of a chronic mucosal inflammation in the absence of polyps.

Healthy controls (n = 48) presented no history of inflammatory nasal complaints and normal findings in the endoscopic examination.

The study was approved by the ethics committee of the medical faculty of Ludwig-Maximilians-University in Munich, Germany, and written informed consent was obtained from all participants.

### Biochemical and immunological methods

Nasal secretions were gained and processed with minor modifications as described by Rasp and co-workers [13]: For the sampling of nasal secretions, small cone-shaped cotton wool pieces (absorbent cotton, Hartmann, Heidenheim/Brenz, Germany) with a length of about 3 cm and a diameter of about 6 mm were used. After positioning the cotton wool pieces in the middle meatus of the nose, they were left in place for 20 min and subsequently centrifuged (+4 °C, 2000*g*) on a sieve for 10 min [14].

Diluted 1:5, all samples were analysed for IL-4, IL-5, IL-10, IL-12, IL-13, IL-17, IL-8, GM-CSF, G-CSF, IFN-γ, MCP-1, MIP-1α, MIP-1β, eotaxin, and RANTES using a human cytokine 17-plex panel (Bio-Plex Cytokine Assay, Bio-Rad Laboratories, Hercules, California). This cytokine assay uses fluorescently-addressed polystyrene beads with conjugated capture antibodies directed to the aforesaid cytokines. After washing, a fluorescently marked detection antibody builds an immunoassay with the cytokine. For analysis, two lasers excite the fluorochromes: one for classifying each bead, the other for quantifying the amount of analyte bound [15]. The detection threshold was 0.5 pg/ml.

ECP and tryptase were measured by ELISA (UniCAP-FEIA, Phadia, Freiburg, Germany). Detection levels were 10 ng/ml for ECP, and 5 ng/ml for tryptase.

### Statistics

SigmaPlot for Windows version 11.0 software (Systat Software, San José, California, USA) was utilised for statistical evaluation and graphical presentation. As all data failed normality testing (Shapiro–Wilk), the Kruskal–Wallis one way analysis of variance (ANOVA) on Ranks was used, testing for statistically significant difference in the median values among the three groups. To isolate the group or groups that differ from the others, the all pairwise multiple comparison procedures (Dunn’s Method) was used in the following step. To reduce the false discovery rate, the method of Benjamini and Yekutieli was used [16]. Data are given as median and range. For graphic presentation, data are displayed in a box plot with the median (horizontal line within the box), the 25th and 75th percentile (boundary of the box), and the 10th and 90th percentile (whiskers above and below the box). Significances are graphically represented between the corresponding plots: * indicates p value <0.05, ** p value <0.01, and *** p value <0.001.

## Results

In total, 141 participants were included in this study, 45 people suffering from NP (28 males, 17 females; mean age 42 ± 15 years), 48 suffering from CRSsNP (18 males, 30 females; mean age 42 ± 15 years) and 48 healthy subjects (18 males, 30 females; mean age 40 ± 16 years).

**Fig. 1:**
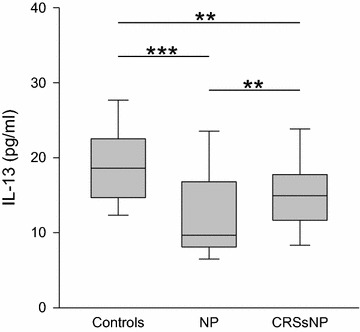
Levels of IL-13 in nasal fluid in controls, NP and CRSsNP: *box plots* of the levels of IL-13 in nasal secretion is shown. IL-13 is significantly decreased in NP compared to both CRSsNP and the controls. Moreover, IL-13 is decreased in CRSsNP compared to the controls. **p < 0.01, ***p < 0.001

The levels of T_H_2 related cytokines presented an inhomogeneous picture (Table 1). For IL-4, the three groups showed no significant differences. The level of IL-5 was increased in NP in comparison to CRSsNP, while a comparison between either of both groups of chronic rhinosinusitis versus controls revealed no differences. As shown in Fig. 1, CRSsNP (median 15 pg/ml, range 2–92 pg/ml; p < 0.01 vs. controls and vs. NP) as well as NP (median 10 pg/ml, range 4–62 pg/ml; p < 0.001 vs. controls) presented reduced amounts of IL-13 (controls: median 19 pg/ml, range 10–32 pg/ml).

**Table 1:** Cytokine levels in nasal fluid in healthy controls, NP and CRSsNP participants

	IL-4	IL-5	IL-8	IL-13	Eotaxin	GM-CSF	RANTES	MCP-1	MIP-1α
Controls	7 0–32	5 1–238	1310 189–42,868	19 10–32	45 0–154	32 0–137	9 0–259	66 17–401	0 0–113
NP	7 0–17	10 0–500	1851 0–265,037	10 4–62	75 0–422	27 0–112	14 0–563	117 13–3867	6 0–60
CRSsNP	5 0–30	3 1–1831	1877 0–1,384,113	15 2–92	49 0–339	42 0–132	35 0–426	89 0–1676	0 0–673
P values									
NP—Con	n.s. (0.822)	n.s. (0.070)	n.s. (0.562)	*s. (<* *0.001)*	n.s. (0.114)	n.s. (0.286)	n.s. (0.447)	*s. (<* *0.001)*	*s. (<* *0.001)*
CRSsNP—Con	n.s. (0.133)	n.s. (0.025)*	n.s. (0.291)	*s. (<* *0.01)*	n.s. (0.361)	n.s. (0.456)	*s. (<* *0.050)*	n.s. (0.028)*	n.s. (0.028)*
NP—CRSsNP	n.s. (0.213)	*s. (<0.001)*	n.s. (0.887)	*s. (<* *0.01)*	n.s. (0.315)	n.s. (0.148)	n.s. (0.079)	n.s. (0.024)*	n.s. (0.037)*

Concentrations are given in pg/ml. Data are presented as median (upper line) and range (lower line). To control the false discovery rate, we used the method of Benjamini and Yukatieli. Thus, values marked with ‘*’ are regarded as non-significant despite p < 0.05

*n.s.* not significant; *s*. significant

**Fig. 2:**
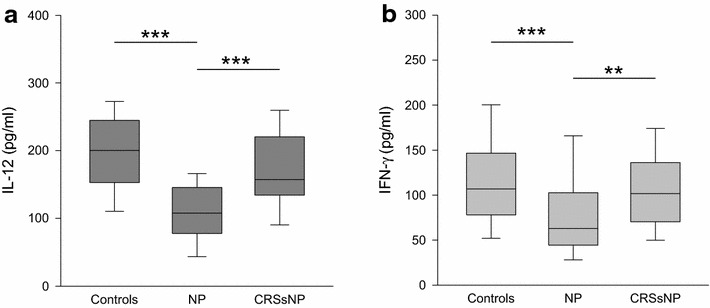
Levels of IL-12 and IFN-γ in nasal fluid in controls, NP and CRSsNP: *box plots* of the levels of IL-12 (**a** *dark grey*) and IFN-γ (**b** *light grey*) in nasal secretion are shown. IL-12 as well as IFN-γ are significantly decreased in NP compared to both CRSsNP and the controls. **p < 0.01, ***p < 0.001

Compared to the controls and CRSsNP, the quantities of T_H_1 associated cytokines IL-12 (Fig. 2a), as well as IFN-γ (Fig. 2b) were decreased in NP (IL-12: median 108 pg/ml, range 17–211 pg/ml, p < 0.001 vs. controls and vs. CRSsNP; INF-γ median 63 pg/ml, range 0–308 pg/ml, p < 0.001 vs. controls and p < 0.01 vs. CRSsNP). CRSsNP (IL-12: median 158 pg/ml, range 60–318 pg/ml; INF-γ median 102 pg/ml, range 0–683 pg/ml) did not differ from the controls (IL-12: median 200 pg/ml, range 59–358 pg/ml; INF-γ median 107 pg/ml, range 34–551 pg/ml).

**Fig. 3:**
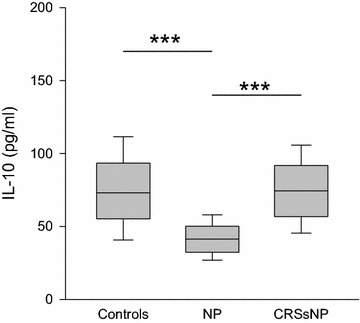
Levels of IL-10 in nasal fluid in controls, NP and CRSsNP: *box plot* of IL-10 levels in nasal secretion is shown. IL-10 is significantly decreased in NP compared to the controls as well as to CRSsNP. ***p < 0.001

Likewise, IL-10 (Fig. 3), a T_reg_ related cytokine, was decreased in NP (median 41 pg/ml, range 8–72 pg/ml) compared to controls (median 73 pg/ml, range 31–158 pg/ml; p < 0.001) as well as to CRSsNP (median 74 pg/ml, range 20–118 pg/ml; p < 0.001).

**Fig. 4:**
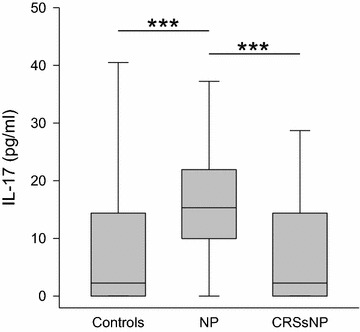
Levels of IL-17 in nasal fluid in controls, NP and CRSsNP: *box plot* of IL-17 levels in nasal secretion is shown. IL-17 is significantly increased in NP compared to both the controls and CRSsNP. ***p < 0.001

In contrast to these diminished cytokine levels, the T_H_17 respective cytokine IL-17 (Fig. 4) was elevated in nasal secretions of NP patients (median 15 pg/ml, range 0–105 pg/ml) in comparison to controls (median 2 pg/ml, range 0–320 pg/ml; p < 0.001) and to CRSsNP (median 2 pg/ml, range 0–146 pg/ml; p < 0.001).

**Fig. 5:**
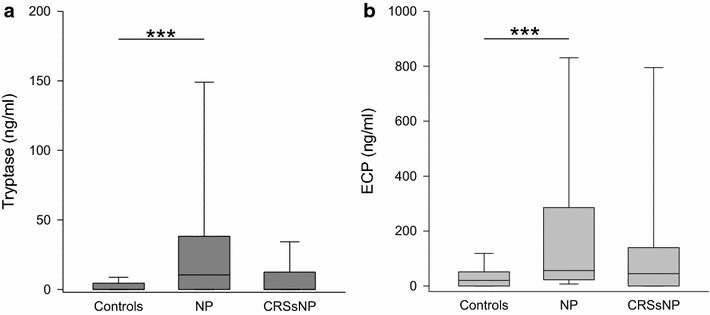
Levels of tryptase and ECP in nasal fluid in controls, NP and CRSsNP: *box plots* of the levels of tryptase (**a** *dark grey*) and ECP (**b** *light grey*) in nasal secretion are shown. Tryptase and ECP are significantly elevated in NP compared to controls. ***p < 0.001

Mast cell activation was seen in NP patients by elevated levels of tryptase in nasal secretion, as indicated in Fig. 5a (NP median 11 pg/ml, range 0–75 pg/ml; controls: median 0 pg/ml, range 0–94 pg/ml; CRSsNP median 0 pg/ml, range 0–75 pg/ml; p < 0.001 vs. controls). Additionally, ECP (Fig. 5b), a marker of eosinophil activation, was increased in NP (NP median 56 pg/ml, range 0–1000 pg/ml; controls: median 20 pg/ml, range 0–467 pg/ml; CRSsNP median 45 pg/ml, range 0–1000 pg/ml; p < 0.001) while the quantity of eotaxin in nasal discharge showed no statistically significant differences among groups (Table 1).

**Fig. 6:**
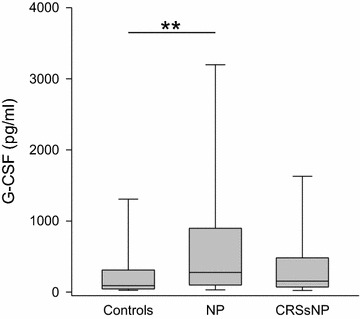
Levels of G-CSF in nasal fluid in controls, NP and CRSsNP: *box plot* of G-CSF levels in nasal secretion. G-CSF is significantly elevated in NP compared to controls. **p < 0.01

Neutrophil associated factors such as IL-8 (Table 1) partially showed a non-significant elevation in nasal secretion from patients with chronic rhinosinusitis either with or without nasal polyps. While in NP G-CSF (Fig. 6) was increased threefold over the controls (NP median 277 pg/ml, range 0–9802 pg/ml; controls: median 90 pg/ml, range 9–7962 pg/ml; CRSsNP: median 155 pg/ml, range 0–8611 pg/ml; p < 0.01), levels of GM-CSF (Table 1) were not different among groups.

**Fig. 7:**
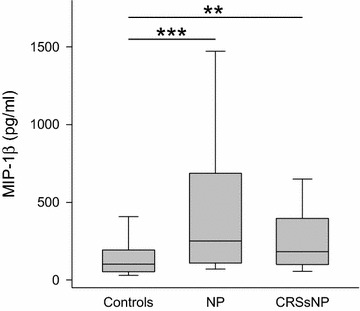
Levels of MIP-1β in nasal fluid in controls, NP and CRSsNP: *box plot* of MIP-1β levels in nasal secretion. MIP-1β is significantly elevated in NP and CRSsNP compared to controls. **p < 0.01, ***p < 0.001

Quantities of chemoattractant proteins were increased in chronic rhinosinusitis. MCP-1 and MIP-1α were significantly elevated in NP only (Table 1). Irrespective of the existence of nasal polyps, levels of MIP-1β (Fig. 7) were significantly increased in NP (median 251 pg/ml, range 12–2088 pg/ml; p < 0.001) as well as in CRSsNP (median 182 pg/ml, range 0–5296 pg/ml; p < 0.01) over controls (median 103 pg/ml, range 0–2049 pg/ml). Concerning RANTES, a statistically significant increase was found only in CRSsNP compared to the controls, whereas levels in NP did not differ from the other groups (Table 1).

## Discussion

This study is part of an extensive project, aiming for distinct cytokine patterns in chronic nasal diseases. CRS seems to be a heterogeneous group of diseases presenting not only different phenotypes like CRS with or without nasal polyps but also consisting of diverse endotypes. New therapeutic approaches with biologic agents are currently in development [17]. These new approaches necessitate patient selection by biomarkers. To determine this reason, there is demand for tools helping to define endotypes as well as to select suitable patients for therapies with anti-cytokine antibodies. Bio-Plex Cytokine Assay in nasal secretion could be such a tool as collection of nasal discharge is an easy procedure harmless to the patient, and the assay is simple to perform. Thus, it constitutes a methodological approach possibly applicable in clinical routine. We therefore have already analysed cytokines in nasal secretions of patients with allergic rhinitis in a true-to-life clinical setting as a first step [18]. In the present study, we measured the amount of cytokines in nasal fluid of participants suffering from NP or CRSsNP as well as healthy controls. The aim of the current study was to investigate whether in CRS with or without nasal polyps, representative cytokines in nasal discharge show distinct patterns proving the used methodology helpful for endotyping inflammatory nasal diseases. In the long term, we aim for providing easily accessible biomarkers allocating patients to specific endotypes and therapies.

IL-4, IL-5, and IL-13 are usually regarded as T_H_2 cytokines, being predominantly involved in the humoral immune response. These cytokines are not only produced by T_H_2 lymphocytes but also by other cells involved in this response pattern, such as plasma cells, mast cells, and eosinophils [10, 19]. For IL-4, we did not find any differences between the three groups, which is in accordance with previous findings [20]. However, other authors observed an elevation in nasal secretions in NP, and a correlation between IL-4 levels and the patients’ CT scores was described [21, 22]. IL-4, as well as IL-13, supports the expression of a T_H_2 inflammatory pattern by modulating lymphocyte differentiation, inducing IgE production, and facilitating eosinophil infiltration by the up-regulation of chemoattractants and adhesion molecules [19, 23]. Moreover, in vitro studies revealed a negative influence of IL-4 on the epithelial integrity in NP [24]. We surprisingly found decreased levels of IL-13 in both CRS groups, contradicting previous reports of an up-regulation of IL-13 mRNA in NP [22, 25]. Using the same control group, we unexpectedly detected decreased levels in allergic rhinitis patients in a previous study [18]. This might imply a methodical error forming the basis of the decreased amounts of IL-13. We are not able to offer a suitable concept for this unexpected result. Concerning IL-5, detected levels in CRSsNP and NP were not significantly different from controls. However, the amount of IL-5 in NP secretions was significantly higher than in CRSsNP. Several authors found elevated levels of this cytokine [25–27]. IL-5 is a hematopoietic growth factor and crucial for the survival and maturation of eosinophils at the site of inflammation [19, 23]. Therefore, it is discussed as a possible therapeutic target in NP and studies with anti-IL-5 monoclonal antibodies show auspicious results [28]. In conclusion, our results indicate a down-regulation of T_H_2 lymphocytes in CRSsNP. Furthermore, they rebut an expected up-regulation in NP and are opposed to the general assumption that the majority of Caucasian NP patients show a T_H_2 pattern of inflammation with elevations of T_H_2-type cytokines.

IL-12 and IFN-γ are indicators of T_H_1 lymphocyte activity. Both cytokines were decreased in NP compared to both the controls and CRSsNP, indicating a down-regulation of T_H_1 cells in nasal polyposis. Others found up-regulated or unchanged levels of IFN-γ and IL-12 in NP and CRSsNP [27, 29]. However, these studies used tissue samples instead of nasal secretions. Both IL-12 and IFN-γ induce a predominantly cellular immune response, involving cytotoxic cells and macrophages. They promote T_H_1 differentiation and counteract T_H_2 and T_H_17 development [30]. Moreover, they influence neutrophil survival as well as epithelial integrity [24, 31]. In a study on mice, IFN-γ expression was shown to be associated with deteriorated olfactory function [32]. Accordingly, this cytokine might be considered a therapeutic target for treating the burdensome reduction of smell in patients suffering from CRS.

IL-10 was used as a reference to the role of T_reg_ in CRS. A decrease was detected in NP which fits the findings of *Kim* et al. who detected impaired migration of regulatory T cells in NPs [33]. This points to a derogated immunomodulation in the mucosa of NPs. Furthermore, the level of IL-17 was sevenfold higher in NP than in the controls or CRSsNP. IL-17 is characteristic for T_H_17 lymphocytes and a proinflammatory cytokine affecting neutrophils and eosinophils [34, 35]. Data on IL-17 is still ambiguous. While elevated levels have been described in Chinese NP patients, studies on Caucasians reveal conflicting results, ranging from elevated to reduced amounts [12, 25, 36]. Thus, further research on this topic might be needed. In conjunction with the aforesaid results, we state that a relative ascendancy of T_H_2 over T_H_1 as well as an up-regulation of T_H_17 was seen in NP while an impaired function of T_reg_ suggests itself in this disease entity. CRSsNP, however, showed normal quantities of all cytokines except for decreased levels of the T_H_2 cytokine IL-13. Our results argue for a more severe inflammation in NP, whereas the inflammation in CRSsNP was only weakly depicted in nasal secretions.

Eosinophilic inflammation has frequently been described in the nasal mucosa of patients suffering from NP. As mentioned, IL-5, a cytokine inducing survival and activation of eosinophils was elevated in NP compared to CRSsNP. Another major factor in eosinophilic inflammation is eotaxin. It is up-regulated preferably by T_H_2 and potently attracts eosinophils [10, 37, 38]. Elevated levels were found in the sinunasal mucosa of CRS patients as well as in nasal secretions of NP patients [27, 39]. In our study highest levels were also seen in NP, however differences between the three groups did not reach statistical significance. The levels of ECP, on the other hand, were significantly elevated in NP but not in CRSsNP. ECP is a protein holding antimicrobial as well as modulatory properties [40]. Plenty of reports of elevations of ECP levels in different nasal diseases exist, indicating that ECP is rather a general marker of inflammation than disease-specific [14, 29, 41]. Our results suggest an infiltration of eosinophils into the mucosa of nasal polyps but not into the mucosa of CRSsNP. In allergic rhinitis, mast cells have frequently been investigated, and much is known about their role in the early-phase of allergic reaction [42]. We detected elevated levels of tryptase in nasal secretions of the NP patients. This is in conformity with findings from others describing an increased amount of mast cells and tryptase in mucosal tissue and nasal secretions of NP patients. Further, the level of tryptase in nasal secretions correlated with nasal obstruction and rhinorrhoea [14, 43]. This might suggest a benefit from mast cell targeting medication in NP.

Di Lorenzo and colleagues reported that the levels of tryptase and ECP in NP exceeded those in allergic rhinitis [44]. We compared the levels of these two mediators in NP with the previously reported levels in allergic rhinitis (AR) [18]. For ECP and tryptase, the levels in seasonal AR were twice as high as in NP, while the values in perineal AR were slightly lower than in NP. However, in contrast to the findings of Di Lorenzo and co-workers, in our study, the differences between the levels in NP and AR did not reach statistically significance. Di Lorenzo et al. gained their samples by nasal lavage while we used the cotton wool method. ECP release was found to be higher in polyps than in the lower turbinate of NP patients [45]. Probably, the amount of ECP and tryptase would be higher under assured placement of the cotton wool pieces on the polyp. This might explain the difference to Di Lorenzo’s results.

Neutrophil infiltration has been seen in both CRSsNP and NP [46]. In order to get indication of neutrophil attraction, we measured the levels of IL-8 and detected elevated amounts in both CRS groups but not reaching a level of significance. Others report a more pronounced increase of IL-8 in NP [47, 48].

The colony-stimulating factors delay neutrophil death [31]. While G-CSF influences proliferation and differentiation of neutrophil progenitor cells as well as the function of mature neutrophils, GM-CSF often appears in the context of recruitment, activation, and survival of eosinophils [38, 49]. Concerning G-CSF, we found levels threefold higher in NP than in controls. GM-CSF was in a normal range in the nasal secretions in CRS, irrespective of nasal polyps, as opposed to elevations described in tissue samples of NP patients [50]. In summary, we saw no definite evidence of increased neutrophil attraction by IL-8, but elevated levels of G-CSF in NP might indicate a role of this type of granulocyte in polyposis.

In addition, different chemokines were examined. RANTES was elevated in CRSsNP but not in NP, others reported increased levels of RANTES in tissue samples of polyps [50, 51]. RANTES is known to attract eosinophils, basophils and mast cells, and is present in nasal secretions during ongoing infection [38, 52, 53]. Plasma levels of RANTES have been found to correlate with disease severity [54]. In contrast to RANTES, we found MCP-1 to be elevated in NP. MCP-1 attracts different inflammatory cells, among them monocytes and T cells. In CRS, increased amounts of MCP-1 have been reported in nasal secretions as well as in nasal mucosa biopsies [29, 55].

Two other chemokines, MIP-1α and MIP-1β, are structurally related proteins, with 68 % of their amino acids being identical [56]. Produced by a host of inflammatory cells, they both have a number of cellular targets, such as monocytes and dendritic cells. However, only MIP-1α is ascribed to attract granulocytes [56, 57]. In our study, increased amounts of MIP-1α were detected in polyposis patients while being undetectable in the majority of the CRSsNP patients and controls. MIP-1β, on the other hand, showed elevated levels in both CRSsNP and NP. Peric and co-workers found a correlation between MIP-1α levels in nasal secretions and endoscopic and CT scores in NP [57]. Moreover, MIP-1α gene expression was elevated in patients with early recurrence of polyps after surgery over those being treatment-responsive [50]. Further research is needed to evaluate the diagnostic and prognostic utility of this chemokine in CRS.

In conclusion, the evaluation of the chemokines and growth factors in the present study revealed an elevation of G-CSF, MCP-1, MIP-1α, and MIP-1β in NP, while CRSsNP shows increased levels of RANTES and MIP-1β only. We conclude that a number of different inflammatory cells are involved in NP and inflammation is more pronounced in NP than in CRSsNP.

## Conclusions

Colleagues from Belgium recently emphasised in their review “Emerging biologics for the treatment of chronic sinusitis”: “The greatest challenge for the future is to define the different endotypes of CRSwNP using easily accessible biomarkers to select the patients who have the best chance of a positive therapeutic response to innovative approaches.” [58]. With the present study, we tried to take a closer look exactly on this topic evaluating cytokine profiles in participants suffering from CRS with or without nasal polyps.

Overall, we found a more pronounced inflammatory profile in NP than in CRSsNP. IL-5, IL-10, IL-12, IL-17, and IFN-γ represent a disequilibrium of T cells in NP, and ECP, tryptase, G-CSF, MCP-1, MIP-1α, and MIP-1β depict the activation of various inflammatory cells in this disease entity. CRSsNP participants, on the other hand, did not differ much from healthy individuals. Merely RANTES and MIP-1β seem to be suitable mediators to distinguish between CRSsNP and healthy individuals. As we did not detect any significant differences between the three groups for IL-4, IL-8, GM-CSF, and eotaxin, we conclude that these mediators are not of distinctive function in chronic rhinosinusitis.

In the long term, we aim to evaluate multiplex-analyses of cytokines in nasal discharge being a suitable diagnostic tool for the “endotyping” of patients with chronic sinonasal diseases. To us, this is a crucial step for selection of patients with regard to a therapy with biologic agents, especially anti-cytokine antibodies. The sampling of nasal secretions is an easily performable and non-invasive method and could benefit many patients if established as a diagnostic and prognostic tool. However, further research regarding suitable indicators of different nasal diseases and the establishment of norm values is needed to attain this goal. Thus, therapies tailored to the individual patient’s needs should become accessible in the future.
